# The Role of Extracellular-Vesicle-Derived miRNAs in Postoperative Organ Dysfunction in Neonates and Infants Undergoing Congenital Cardiac Surgery: An Exploratory Study

**DOI:** 10.3390/ijms26083837

**Published:** 2025-04-18

**Authors:** Fahd Alhamdan, Wiriya Maisat, LeeAnn Higgins, Yue Chen, Juan Ibla, Koichi Yuki

**Affiliations:** 1 Department of Anesthesiology, Critical Care and Pain Medicine, Cardiac Anesthesia Division, Boston Children’s Hospital, Boston, MA 02115, USA; fahd.alhamdan@childrens.harvard.edu (F.A.); pians17@gmail.com (W.M.); juan.ibla@childrens.harvard.edu (J.I.); 2 Department of Anaesthesia, Harvard Medical School, Boston, MA 02115, USA; 3 Department of Immunology, Harvard Medical School, Boston, MA 02115, USA; 4 Broad Institute of MIT and Harvard, Cambridge, MA 02142, USA; 5 Department of Anesthesiology, Faculty of Medicine Siriraj Hospital, Mahidol University, Bangkok 73170, Thailand; 6 Department of Biochemistry, Molecular Biology and Biophysics, University of Minnesota, Minneapolis, MN 55455, USA; higgi022@umn.edu (L.H.); yuechen@umn.edu (Y.C.)

**Keywords:** congenital heart disease, organ dysfunction, neutrophil extracellular trap, complement, miRNA, extracellular vesicle

## Abstract

Despite significant advancements in medical and surgical care, the morbidity and mortality rates of neonates and infants undergoing congenital cardiac surgery remain high. To identify new pathomechanisms associated with postoperative organ dysfunction, extracellular vesicles (EVs) were isolated from plasma from neonates and infants with or without organ dysfunction at three different time points around congenital cardiac surgery, and the EV miRNA expression profiles in the plasma were analyzed. A clear distinction was observed between the organ dysfunction (OD) and non-organ dysfunction (NOD) groups based on their EV miRNA expression profiles. Apoptosis and proinflammatory pathways were consistently upregulated across all time points in the OD group. Complement and coagulation cascades unexpectedly displayed downregulation at the end of the surgery in the OD group, which was verified further at the proteomic level in an independent patient cohort. The neutrophil extracellular trap (NET) formation was enhanced in the OD group across all time points compared to that in the NOD group. As NETs are known to consume complement components, these observed events might be interconnected. A feature selection machine learning method identified miR-200b-5p, miR-4800-5p, miR-363-3p, and miR-483-5p as robustly linked to organ dysfunction following congenital cardiac surgery (accuracy score = 9; SD in accuracy = 0.3162). In conclusion, our study suggested that neonates and infants with postoperative organ dysfunction were associated with enhanced NET formation and complement consumption.

## 1. Introduction

While the overall outcomes of patients with congenital heart diseases (CHDs) have significantly improved, neonates and infants undergoing cardiac surgery remain associated with high morbidity and mortality. According to the Kids’ Inpatient Database (KID), from 2000 to 2006, the in-hospital mortality rate in neonates and infants was 6.9%, while the mortality rates in the age groups of 1–5, 6–12, and 13–17 years were 1.28%, 0.67%, and 0.83%, respectively [1]. An analysis using the Society of Thoracic Surgeons (STS) Congenital Heart Surgery Database from 2014 to 2017 showed a median mortality of 8.1% and major complications in 25.7% of neonates, indicating that the younger population remains vulnerable [2]. A recent publication on a single center’s experience from 2013 to 2022 also showed very high mortality in this cohort (14.7%) [3]. The clinical risk factors associated with morbidity and mortality in neonates and infants undergoing cardiac surgery include a longer cardiopulmonary bypass (CPB) time [4] and greater blood transfusion requirements [5,6,7]. Thrombotic complications and subsequent organ dysfunction/failure are responsible for worse outcomes [8]. Therefore, it is critical to understand the mechanisms of how they develop thrombotic complications and organ dysfunction/failure.

Extracellular vesicles (EVs) are membrane-enclosed spheres that carry a diverse cargo of functional proteins, miRNAs, lipids, and other biochemical molecules [9]. The pleitropic role of EVs has been described in a variety of diseases [10,11,12,13]. This can be attributed to their capability to mediate the communication between nearby and distal cells [14].

miRNAs are endogenous 18- to 24-nucleotide single-stranded noncoding RNA molecules that govern regulatory functions by targeting mRNAs for their cleavage or translational repression [15,16]. The involvement of miRNAs in a number of diseases has been reported [17,18]. A growing body of literature indicates that a group of miRNAs is responsible for inflammation and thrombosis [19,20,21]. Extracellular miRNA mainly exists in the EVs, and most of the functions of miRNAs occur through the intercellular transmission of EVs [22,23]. Thus, we compared the EV miRNA profiles in neonates and infants undergoing congenital cardiac surgery on CPB who developed organ dysfunction with those who did not to delineate their miRNA signatures and the related biological mechanisms. We also performed proteomics to supplement our findings. This helped us to identify new pathomechanisms associated with organ dysfunction and/or thrombosis following congenital cardiac surgery.

## 2. Results

### 2.1. The Plasma EV miRNAs from Neonates and Infants with Postoperative Organ Dysfunction Distinguished Those from Individuals Without Organ Dysfunction

We sought to identify plasma EV miRNAs associated with postoperative organ dysfunction as new pathomechanisms in pediatric congenital heart surgery and utilize plasma proteomics to validate our findings. The characteristics of all of the neonates and infants enrolled in this study are shown in Table 1. The patients who developed postoperative organ dysfunction (OD) had longer operating times and CPB times and larger volumes of intraoperatively transfused salvaged red blood cells (RBCs) and platelets, consistent with the previously published data [4,5,6,7]. The OD group had longer mechanical ventilation (MV) times, ICU stays, and hospital stays. Consistently with this, the PELOD-2 scores were higher in the OD group at ICU admission and on postoperative day 1.

**Figure 1:**
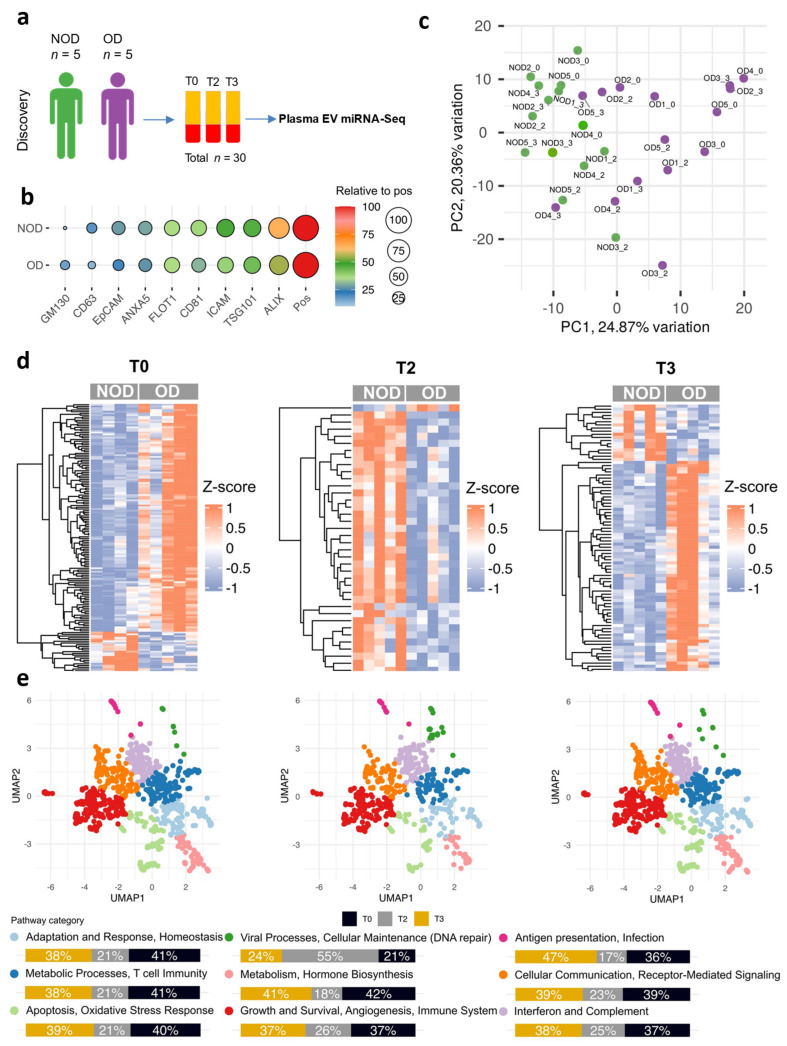
Expression profiles of plasma EV miRNAs during pediatric congenital cardiac surgery. (**a**) A schematic representation of the study design. Organ dysfunction = OD (*n* = 5) and non-organ dysfunction = NOD (*n* = 5). T0: after anesthesia induction (baseline); T2: upon admission to the ICU; and T3: on postoperative day 1. (**b**) The EV proteomic antibody array measuring the levels of eight EV protein markers. Pos, Positive control; GM130, cellular contamination marker. (**c**) A principal component analysis (PCA) of the expression profiles of the plasma EV miRNAs in the OD and NOD individuals at the three time points. (**d**) Heatmaps showing the expression patterns of differentially expressed EV miRNAs between patients with postoperative OD and those without it (NOD) across the three time points. Each row represents a miRNA, and each column represents a sample. (**e**) Uniform Manifold Approximation and Projection (UMAP) illustrates the enrichment in biological pathways predicted from the miRNA target genes (*p* < 0.05). Each dot represents a biological pathway; the colors represent pathway categories (groups of functionally related pathways), and the accompanying bar plots show the proportion of these categories across the three time points.

For the plasma EV miRNA analysis, we included five neonates and infants who underwent congenital cardiac surgery and developed postoperative organ dysfunction (Figure 1a and Table S1). Five individuals in the same age range who did not develop any postoperative organ dysfunction (NOD) served as a comparison group to the OD group.

We isolated EVs from plasma from all individuals at the following three time points: T0: at baseline; T2: upon admission to the ICU; and T3: on postoperative day 1. Subsequently, we characterized the size and protein markers of the EVs and performed EV small RNA-sequencing.

To ascertain the validity of the isolated EVs, we examined a panel of established EV protein markers (Figure 1b and Figure S1a). All markers were detected in both study groups, with ALIX and TSG101 (exosome markers) showing the highest expression. Consistent with this, the NTA revealed a median EV size of less than 150 nm, which fell within the typical range for exosomes [24] (Figure S1b).

Next, we conducted a small RNA-sequencing analysis of the EV miRNA cargo. The principal component analysis (PCA) of the EV miRNA expression profiles demonstrated a notable distinction between the OD and NOD groups (Figure 1c). PCAs of the OD group alone and the NOD group alone demonstrated overlapping EV miRNA expression profiles between T0 and T3 (Figure S2).

To address the differences between OD and NOD at each time point above, we computed the differentially expressed (DE) miRNAs for the three pairwise comparisons (Figure 1d and Table S2). By setting the threshold for the FDR to <0.05, the OD miRNAs revealed 131 upregulated and 24 downregulated miRNAs at T0; 37 upregulated and 1 downregulated miRNA at T2; and 67 upregulated and 18 downregulated miRNAs at T3 compared to the NOD miRNAs. Then, we constructed a Uniform Manifold Approximation and Projection (UMAP) for the enriched biological pathways (*p* value < 0.05) of the target genes for the DE miRNAs (Figure 1e). Although remarkable downregulation in the majority of molecular mechanisms was exhibited at T2, recovery was shown at T3, except for viral processes and cellular maintenance (DNA repair), both of which peaked at T2.

**Figure 2:**
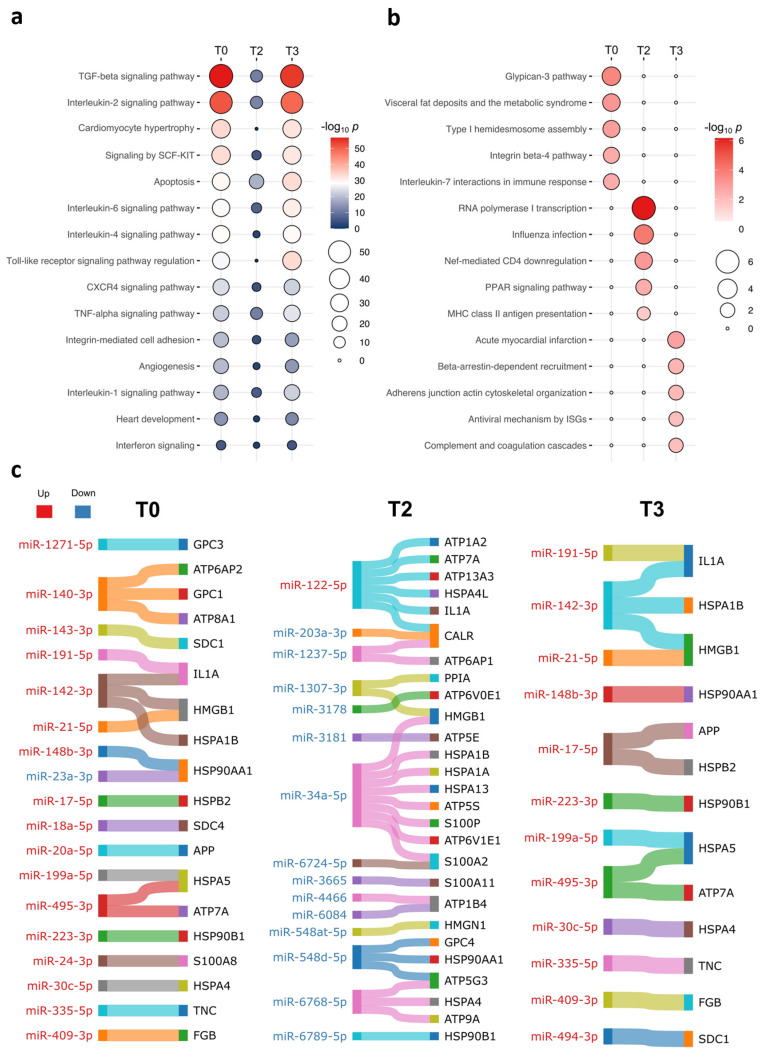
The top 15 unique and shared biological pathways for the plasma EV miRNAs at the three time points during pediatric congenital cardiac surgery. Dot plots exhibiting the top 15 significant biological pathways (*p* < 0.05) of the EV miRNA target genes that were either (**a**) shared or (**b**) uniquely enriched at the three time points during pediatric congenital cardiac surgery. (**c**) Tables of EV miRNA target genes associated with damage-associated molecular pattern (DAMP) molecules at the three time points. Up- and downregulated EV miRNAs are shown in red and blue, respectively. Gene(s) associated with each miRNA are shown on the right.

Next, we inspected the top 15 significant biological pathways shared at all the three time points (Figure 2a). While TGF-β signaling, interleukin-2 signaling, and cardiomyocyte hypertrophy were prominent at T0 and T3, apoptosis and interferon signaling remained stable throughout the three time points. The top five unique pathways enriched at T3 included acute myocardial infarction, β-arrestin-dependent recruitment, and the complement and coagulation system (Figure 2b). These pathways have previously been associated with cardiac complications [25,26].

Apoptosis persisted throughout the cardiac surgery (Figure 2a). It is noteworthy that apoptotic cells have been known to release damage-associated molecular patterns (DAMPs) [27]. Thus, we intended to search for DAMP-coding genes across the DE miRNA target genes [28] (Figure 2c). Higher enrichment in the DAMP-coding genes was exhibited at T2, which were largely targets for downregulated miRNAs.

### 2.2. The Unique DE miRNA Signature at T3 Was Associated with the Complement System

**Figure 3:**
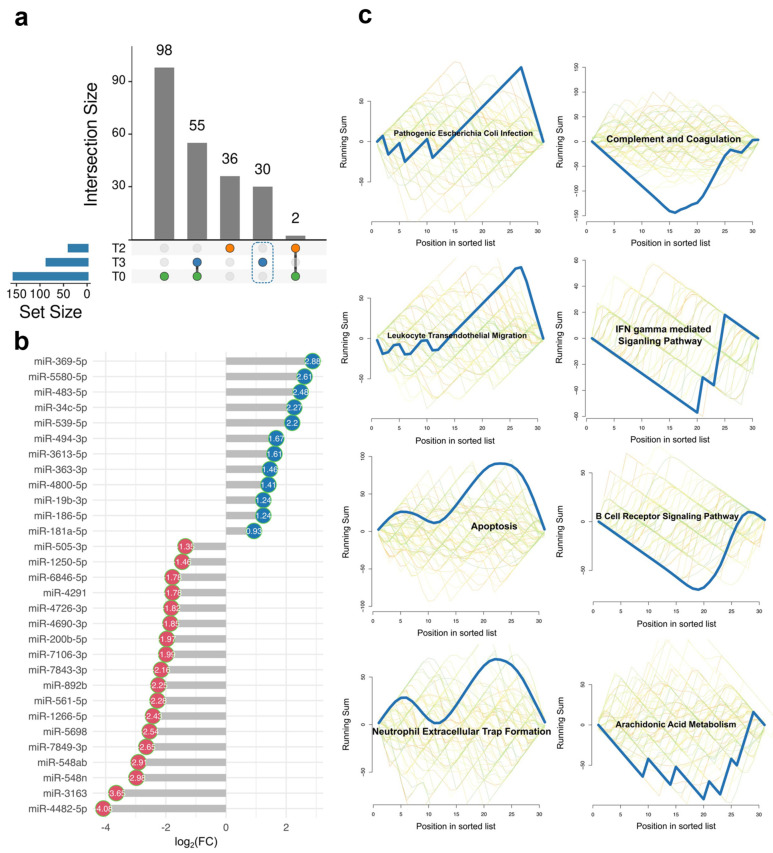
EV miRNAs unique to T3 and their associated biological pathways. (**a**) UpSet diagram showing unique and shared EV miRNAs in OD versus NOD across the three time points during congenital cardiac surgery. (**b**) Dot plot showing the 30 unique EV miRNAs at T3 ordered according to their log_2_(FC) scores. Up- and downregulated miRNAs are shown in blue and red, respectively. (**c**) Gene set enrichment analysis (GSEA) plots of upregulated or downregulated biological pathways associated with the EV miRNAs unique to T3. The simulated background distributions (green to orange lines) and actual enrichment in the respective pathways (blue line) are shown.

So far, we have examined the miRNA profiles in the OD and NOD groups at various time points. Next, we aimed to probe the unique EV miRNAs at T3, as we assumed that they might underpin the mechanism of organ dysfunction. The UpSet diagram depicted 30 DE miRNAs uniquely enriched at T3 (Figure 3a), among which were 12 upregulated and 18 downregulated miRNAs in the OD group compared to those in the NOD group (Figure 3b). Then, we assessed the biological pathways associated with these miRNAs using an enrichment analysis (Figure 3c). Apoptosis, infection, cell migration, and neutrophil extracellular trap (NET) formation were upregulated in OD compared to NOD at T3. The enhanced NET formation in the OD group was consistent with our previous finding [29].

The complement system is reported to undergo activation following CPB due to the direct interaction of the blood components with the extracorporeal circuit [30]. However, our data unexpectedly showed downregulation in the complement system protein levels in OD compared to NOD at T3 (Figure 3c). We additionally probed whether this decline in the complement system protein level already existed at T0. However, the initial pathway enrichment analysis did not identify the complement system (Figure S3), suggesting that it might either have been unaffected or previously activated at baseline in the OD group. Thus, we delved into the complement system protein level patterns across all time points next.

**Figure 4:**
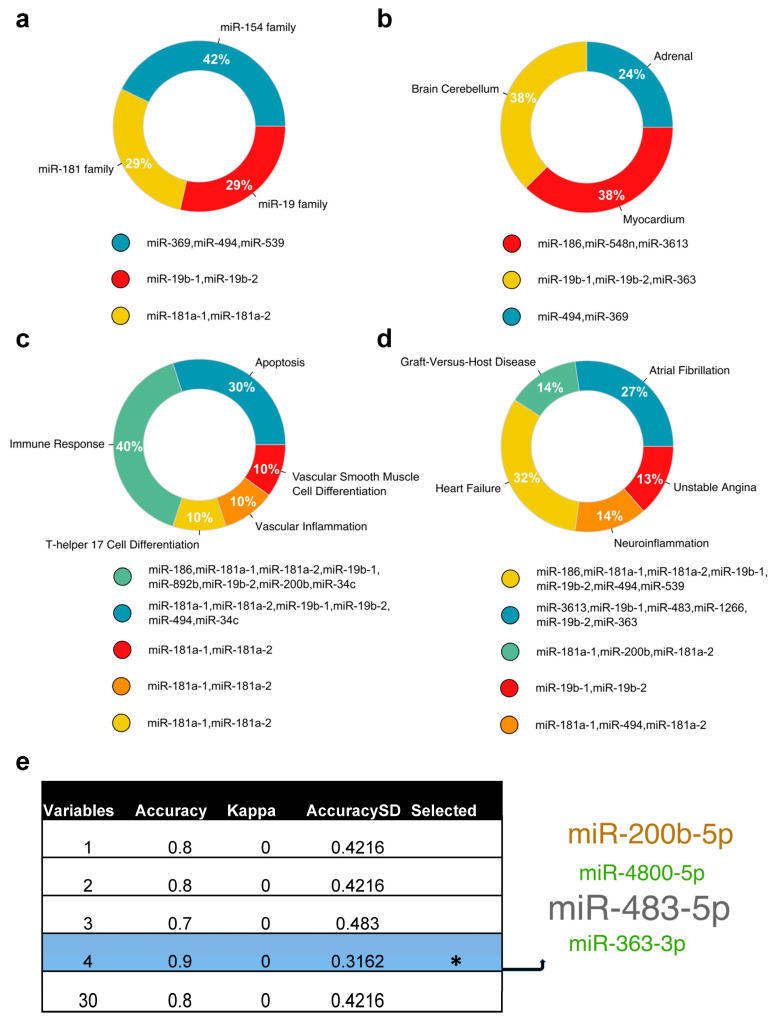
Functional and statistical analyses of the EV miRNAs unique to T3. Donut charts showing the miRNA-based proportions of (**a**) miRNA families, (**b**) tissue specificity, (**c**) biological function, and (**d**) associated diseases with related miRNAs from the 30 unique EV miRNAs at T3. (**e**) A feature selection machine learning method implemented with cross-validation was employed to select a combination of candidate miRNAs (miR-200b-5p, miR-4800-5p, miR-483-5p, and miR-363-3p) with the highest accuracy scores and lowest standard deviation (SD). * denotes highest performance.

To gain a granular view of the unique DE miRNAs at T3, we utilized the TAM 0.2 platform to perform an enrichment analysis for miRNA family, tissue specificity, biological functions, and related diseases (Figure 4a–d) at a significance threshold of *p* < 0.05. The miR-154 family with its three associated miRNAs, namely miR-369, miR-494, and miR-539, represented the most significant family (Figure 4a). The myocardium as well as the brain cerebellum as tissue sources (Figure 4b); immune response, apoptosis, and vascular inflammation as biological functions (Figure 4c); and heart failure and neuroinflammation as related diseases (Figure 4d) might be relevant to organ dysfunction during congenital heart surgery. In addition to the heart, the brain would be an important organ impacted because a period of circulatory arrest was utilized in the group of neonates and infants to repair their cardiac lesions.

To assess the statistical power of the 30 DE miRNAs and select representative candidates, we employed a feature selection machine learning algorithm implemented with cross-validation (Figure 4e). This analysis identified miR-200b-5p, miR-4800-5p, miR-483-5p, and miR-363-3p as the miRNAs with the highest performance, achieving an accuracy of 0.9 and a standard deviation (SD) in accuracy of 0.32.

### 2.3. Delineation of the Complement System Patterns in the Perioperative Period for Congenital Cardiac Patients Using Plasma Proteomics

Since one of the major aspects of the miRNA post-transcriptional regulation machinery is to prevent the translation of RNA into proteins, we sought to examine the plasma proteomic profiles in the perioperative period before pediatric congenital cardiac surgery. Therefore, we enrolled six and four independent neonates and infants with or without organ dysfunction, respectively (Figure S4a and Table S3).

Subsequently, we performed a mass-spectrometry-based proteomics analysis. A pairwise comparison of OD versus NOD revealed 32 upregulated and 33 downregulated proteins at T0, 31 upregulated and 36 downregulated proteins at T2, and 32 upregulated and 39 downregulated proteins at T3 (Figure S4b). We additionally depicted the top 10 upregulated and downregulated proteins for each single comparison (Figure S4c). A consensus analysis of the shared pathways demonstrated that the complement and coagulation cascades were upregulated at T0 but displayed a decrease at T2 and T3 in the OD group compared to the NOD group (Figure S5).

**Figure 5:**
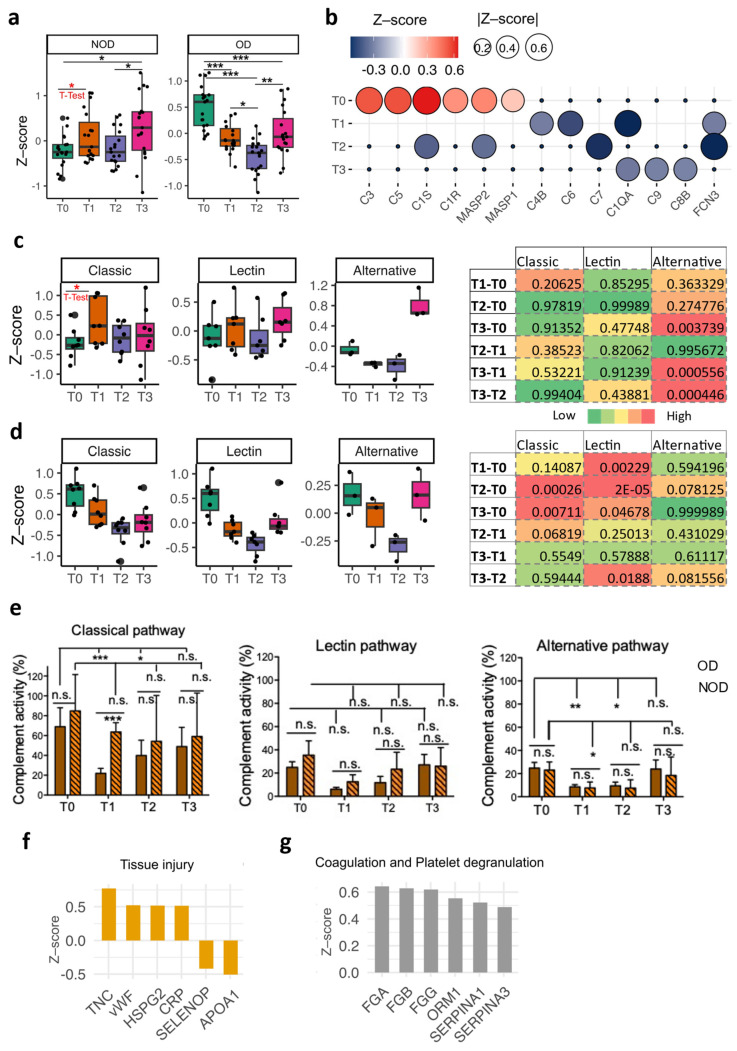
Analysis of complement system patterns in OD and NOD groups. (**a**) Box plots of the complement system patterns based on the Z-scores of their components across the four time points in OD or NOD. (**b**) A dot plot showing the differential expression of the significant complement system components in OD versus NOD compared and at each time point. Box plots displaying the patterns of the three complement system pathways (classic, lectin, and alternative) across the four time points in (**c**) NOD or (**d**) OD, with their associated significance levels on the right side. (**e**) Plasma residual complement activity levels. A one-way ANOVA with Bonferroni’s post hoc analysis was performed. *, **, and *** = *p* < 0.05, *p* < 0.01, and *p* < 0.001, respectively. Bar plots showing Z-scores of proteins associated with (**f**) tissue injury or (**g**) coagulation and platelet degranulation. A one-way ANOVA with Bonferroni’s post hoc analysis and Student’s unpaired *t*-test were used for statistical significance, with *p* values * < 0.05, ** < 0.005, and *** < 0.001. n.s. not significant.

To probe the complement activation caused by CPB [31], we analyzed the pattern of the complement component levels in both the NOD and OD groups at T1, T2, and T3 compared to those at the baseline (T0) (Figure 5a and Table S4). As anticipated, the NOD group sustained higher levels of complement components throughout T2 and T3, following an expected increase at the end of CPB (T1). Conversely, the OD group exhibited a significant decline in its complement component levels at T1. This decline persisted at T2 and remained comparable at T3. This pattern was consistent with the complement system pattern observed in the EV miRNAs. The comparison of the single complement system proteins in the OD group to those in NOD group is depicted in Figure 5b, showing that mannose-binding lectin (MBL)-associated serine protease 1 (MASP1) and MASP2 in the lectin pathway were among the upregulated proteins at the baseline. In line, upregulation in the lectin pathway was observed at baseline (Figure S6).

As complement activation occurs via the three complement pathways (classical, lectin, and alternative), we probed each (Figure 5c–d and Table S4). Our data suggested that the classical and alternative pathways in NOD seemed to drive the overall complement pattern observed perioperatively (Figure 5c). In contrast, all three complement pathways in OD followed a similar tendency to that seen for the global complement pattern (Figure 5a). The lectin pathway seemed downregulated at both T1 and T2 in OD according to the protein levels (Figure 5d). This could be attributed to Ficolin 3 (FCN3), a key upstream activator of the lectin pathway, which showed significant downregulation at these two time points in the OD group compared to that in the NOD group (Figure 5b). We also performed measurements of the activity of each complement pathway. We found that classical- and alternative-pathway-related complements were significantly consumed (Figure 5e). Particularly, classical-pathway-related complements were significantly consumed in the OD group.

The heightened levels of the complement components observed in the OD group at the baseline may be attributed to tissue injury or vascular leakage [32]. Several tissue-injury-associated proteins, including tenascin C (TNC), heparan sulfate proteoglycan 2 (HSPG2), and Von Willebrand factor (VWF) [33,34,35], were upregulated in OD versus NOD at the baseline, while selenoprotein P (SELENOP), apolipoprotein A1 (APOA1), and vascular endothelial protection mediators [36] were downregulated (Figure 5f). This observation was also supported by the upregulation in the inflammation marker C-reactive protein (CRP) at the baseline. An additional cause of this elevated complement level at the baseline may be attributed to difference in the type of upregulated coagulation- and platelet-degranulation-associated proteins in OD versus NOD [37] (Figure 5g).

### 2.4. Cross-Validation of the Complement Signatures Using the Plasma EV miRNAs at T3

**Figure 6:**
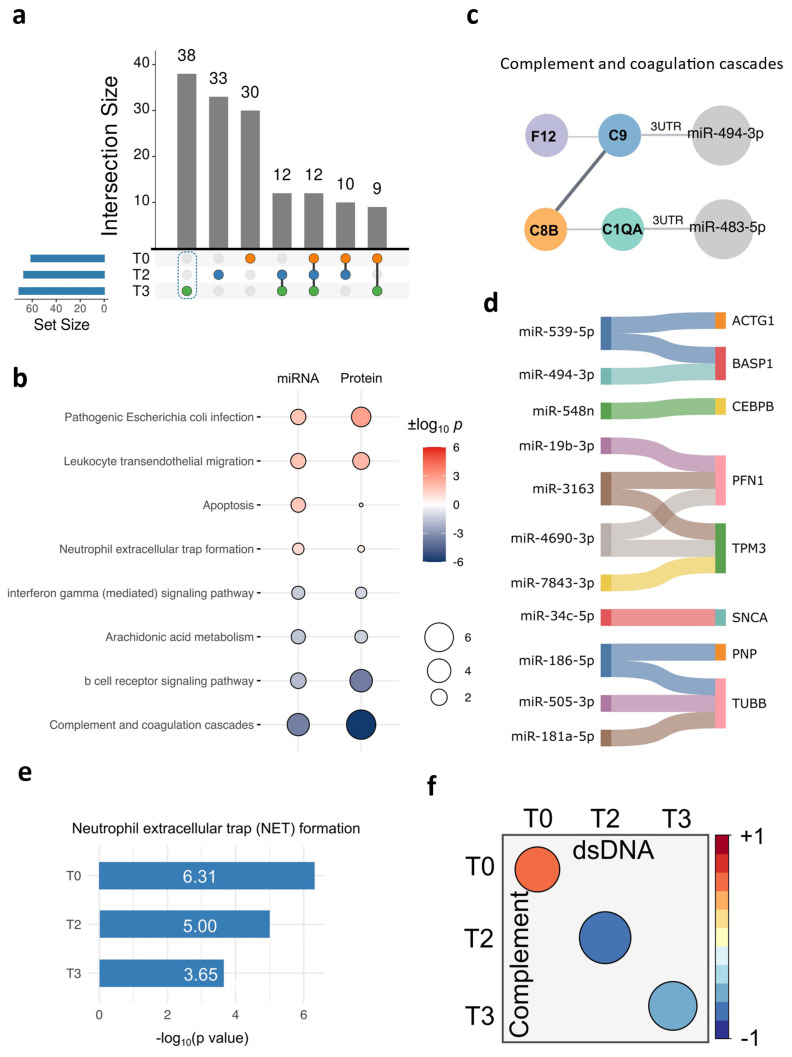
Combined analysis of plasma EV miRNomics and plasma proteomics for the unique T3 signatures. (**a**) UpSet diagram showing unique and shared plasma proteins in OD versus NOD across the three time points during pediatric congenital cardiac surgery. (**b**) Dot plot showing shared biological pathways associated with the 30 unique EV miRNAs and 38 unique plasma proteins at T3. (**c**) A protein–protein interaction analysis of the complement and coagulation cascade components with their targeting EV miRNAs in their untranslated regions (UTRs). (**d**) A Sankey diagram exhibiting the EV miRNAs and their target genes that were uniquely enriched at T3. (**e**) A bar plot of the NET formation enrichment across the three time points in the proteomics data. (**f**) Pearson’s correlation analysis between the plasma dsDNA and complement levels across the three time points.

The downregulation of the complement protein levels at T3 in the OD group observed in the plasma proteomics experiment was consistent with the phenotypes from the miRNA profiles. To explore the possibility that EV miRNAs were at least responsible for the regulation of NET formation and complement activity, we inspected the uniquely enriched DE proteins at T3 (Figure 6a) and examined their associated biological pathways. We then cross-referenced these pathways with those identified from the EV miRNA analysis (Figure 6b). Remarkably, all of the pathways observed in the EV miRNA analysis (Figure 3c) were also enriched in the plasma proteomics data. This indicates potential regulation of the complement activity by the EV miRNAs at T3.

Among the DE miRNAs uniquely enriched at T3, we predicted that miR-494-3p and miR-483-5p targeted proteins/genes associated with the complement and coagulation cascades (Figure 6c). Notably, miR-483-5p was among the four statistically relevant EV miRNAs at T3 in the previous analysis (Figure 4d). The remaining DE miRNAs and their predicted target DE proteins/genes uniquely enriched at T3 are depicted in Figure 6d.

The observed upregulation of NET formation in both datasets might have been a consequence of the complement system activation at baseline [38], and NET formation may potentially have led to the lower complement component levels following CPB. This is because NET formation is known to utilize and potentially consume complement components [39]. Thus, we overlaid the enrichment levels of NET formation across all three time points in the proteomics data and observed the consistent upregulation of NET formation in OD compared to NOD across all time points, with the highest enrichment observed at the baseline (Figure 6e). We additionally evaluated the correlation between the plasma dsDNA concentrations (a NET indicator) [40] and the complement levels across the three time points (Figure 6f). As anticipated, a positive correlation was depicted at T0, while a negative correlation emerged at the later time points (T2 and T3).

**Figure 7:**
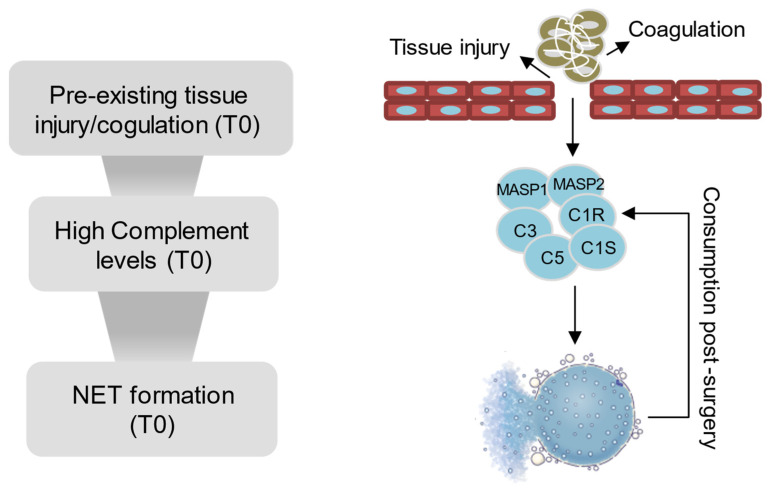
A schematic representation of the different pathomechanisms potentially contributing to organ dysfunction following pediatric congenital cardiac surgery.

Our findings may indicate that pre-existing tissue injury or coagulation and platelet degranulation might have triggered the complement system activation at the baseline (T0). This early complement activation could then at least in part have contributed to the induction of NET formation, a process known to also consume complement components, potentially contributing to the lower complement levels observed post-CPB and -surgery in the OD group (Figure 7).

## 3. Discussion

In our current study, we utilized EV miRNAs to elucidate the mechanisms that might underpin organ dysfunction in neonates and infants undergoing congenital cardiac surgery. By employing an analysis at three time points (pre-surgery, at ICU admission, and on postoperative day 1), we were able to identify a myriad of DE miRNAs in OD versus NOD at each time point. The biological pathways associated with these DE miRNAs, including apoptosis and diverse proinflammatory pathways enriched at all time points, may indicate a pre-existing inflammatory state in the OD group [41]. This notion was evidenced by the elevated CRP levels at T0. Likewise, elevated tissue-injury-associated proteins such as TNC, VWF, and HSPG2, along with decreased levels of protective factors, including SELENOP and APOA1, indicated the presence of endothelial cell injury or vascular leakage.

Focusing mainly on T3, we identified four EV miRNAs—miR-200b-5p, miR-4800-5p, miR-363-3p, and miR-483-5p—that might have contributed to organ injury following congenital cardiac surgery. miR-363-3p has been demonstrated to regulate vascular endothelial injury during acute myocardial infarction (AMI) [42]. Thus, the knockdown of miR-363-3p ameliorated the cellular proliferation of human umbilical vein endothelial cells. Additionally, miR-363-3p promoted apoptosis in cardiomyocytes during cardiomyocyte injury and served as a biomarker for predicting the protective effects of isoflurane on attenuating cardiomyocyte injury [43].

The pathophysiological role of miR-483-5p has been implicated in endothelial cell injury [44]. The downregulation of miR-483-5p in human umbilical vein endothelial cells decreased cell apoptosis and the expression levels of proinflammatory cytokines such as IL-1β and IL-6. This alleviated the endothelial cell injury induced by oxidized low-density lipoprotein (ox-LDL). In addition, high serum levels of miR-483-5p have been utilized to predict postoperative atrial fibrillation following coronary artery bypass grafting [45] and to diagnose acute coronary syndrome (ASC) and predict cardiovascular events (MACEs) in patients undergoing percutaneous coronary interventions (PCIs) [46]. Additionally, miR-483-5p has been shown to prevent neurological impairments after cardiopulmonary resuscitation through targeting TNFSF8 (TNF superfamily member 8) in the AMPK/JNK pathway [47]. This and other studies further confirm the utility of modulating miR-483-5p expression as a potential therapeutic strategy for cardiovascular diseases. This could be achieved through the use of miRNA mimics (to increase its expression) or antagomirs/inhibitors (to decrease its expression). Therefore, experimental research to underpin the role of miR-483-5p in inhibiting organ dysfunction following congenital cardiac surgery is highly recommended. In contrast to miR-363-3p and miR-483-5p, the role of miR-200b-5p and miR-4800-5p in the cardiac surgery setting is not well known. miR-200b-5p has been shown to inhibit tumor progression (ref). miR-4800 also inhibits tumor proliferation (ref).

Complement system levels have been known to increase post-CPB during congenital cardiac surgery [30]. Although this trend was observed in the NOD group in our data, the OD group exhibited a significant decline post-CPB, along with pathological complement elevation at the baseline. This elevation may have been due to tissue injury or coagulation and platelet degranulation [32,37]. The observed upregulation of the MASP1 and MASP2 levels at the baseline in OD, followed by the downregulation of FCN3 at T1 and T2, suggests the involvement of the lectin pathway. A prospective study involving 190 pediatric patients undergoing congenital cardiac surgery with the use of CBP investigated the role of the lectin pathway components in the development of postoperative complications [48]. In said study, individuals with serum FCN3 levels lower than 10.1 µg/mL had a high probability of experiencing systemic inflammatory response syndrome (SIRS) and multi-organ dysfunction (MODS). Meanwhile, high serum MASP1 levels (11.68 µg/mL) were associated with a high mortality rate, suggesting that lectin pathway components may be engaged in the mechanisms of postoperative complications or organ dysfunction. In our study, we showed a significant reduction in classical-complement-mediated activation in the OD group during CPB (T1). C1QA is part of the classical complement pathway. As aforementioned, miR-483-5p is involved in various cardiac diseases and has been tested as a therapeutic target. Our data demonstrated that miR-483-5p could target the complement system, particularly C1QA at T3. It has been reported that C1QA has an inhibitory effect on the formation of left ventricular hypertrophy and thus a protective effect against myocardial injury during cardiac surgery [49]. Given these findings, investigating the parallel roles of miR-283-5p and C1QA in the development of postoperative complications holds significant promise.

The complement system is able to induce NET formation [38]. Our data revealed that C3 was among the upregulated complement components at the baseline. In line with this, C3 has been shown to promote neutrophil infiltration and directly induce NET formation [50]. This process could contribute to the development of remote organ dysfunction. Another recent study by us further highlights the crucial role of NET formation in the development of postoperative organ dysfunction [29]. NET formation offers a potential explanation for the lower complement levels observed post-CBP. This is because NET formation has been reported to consume complement components [39]. In line with this, others have reported the formation of NETs during cardiac surgery (ref). The CPB circuit was primed using RBCs and FFP in the same way for both the OD and NOD groups. RBCs, FFP, platelets, and cryoprecipitates contain complement components in their products [51,52]. While the NOD group showed an increase in its complement levels at T1, the OD group showed a decrease, suggesting that this reduction could be attributed to complement consumption. The complement levels were comparable between T1 and T2 in the NOD group. In contrast, a further reduction in their levels was seen from T1 to T2 in the OD group. In the post-CPB phase, the patients received salvaged RBCs. Given that the complement components are largely removed by washing salvaged RBCs [53,54], complement levels would be diluted by an intraoperative salvaged RBC transfusion. However, platelet and cryoprecipitate transfusions replete complements. The platelet/salvaged RBC ratio in the OD vs. the NOD group in the post-CPB phase was (16.7/32.7):(10.9/16.3) = 1.2:1. Similarly, the cryoprecipitate/salvaged RBC ratio in the OD vs. the NOD group in the post-CPB phase was (7.7/32.7):(4.1/16.3) = 0.94:1. The ratios were comparable between OD and NOD. Because the complement levels in NOD between T1 and T2 were similar, the reduction in the complement levels at T2 from T1 in OD could also be suggestive of their consumption.

We acknowledge the limited number of individuals enrolled in this study and the lack of mechanistic experiments. While our study was exploratory in nature, our findings of enhanced NETs in the OR group from the miRNA profiles were in line with the findings of our previous study in a larger cohort, where we demonstrated NETs using traditional myeloperoxidase (MPO)–DNA complex level measurements [29]. However, our data highlight the critical role of EV miRNA cargo and post-transcriptional regulation in biological processes, including the complement system and NET formation, and subsequent postoperative complications and organ dysfunction in congenital heart disease patients.

## 4. Materials and Methods

### 4.1. Patient Enrolment and Clinical Data Collection

This single-center prospective study was approved by the Institutional Review Board at Boston Children’s Hospital. Neonates and infants scheduled for congenital cardiac surgery on CPB were included in this study We excluded patients who did not necessitate CPB, had an active infection preoperatively, were on chronic steroid therapy, had a history of malignancy, or required mechanical ventilatory support or inotropic support just prior to their surgery. Patients were enrolled from 31 May 2022 to 22 February 2023. Written consent was obtained from their parents or legal guardians. Demographic information, diagnosis, type of procedure, laboratory values, CPB details, type and volume of transfused blood products, and postoperative complications were extracted from their electronic medical records. Postoperative complications were defined as the presence of organ dysfunction/failure and/or thrombosis. Due to the absence of a standardized definition for organ dysfunction following congenital cardiac surgery, we adopted the previously published criteria [55,56] which we previously used [29]. Briefly, each organ dysfunction was defined as follows: (1) Cardiovascular dysfunction was defined as (i) the requirement for a vasoactive agent and/or (ii) elevated lactic acid; (2) respiratory dysfunction was defined as (i) the need for postoperative mechanical ventilation > 5 days and/or (ii) arterial oxygen tension/a fraction of inspired oxygen (PaO_2_/FiO_2_) < 300; (3) neurological dysfunction was defined as (i) the development of a new intracranial infarct or hemorrhage and/or (ii) evidence of hypoxic–ischemic injury through clinical examination or imaging; (4) hepatic dysfunction was defined as bilirubin of >2 mg/dL; (5) renal dysfunction was defined as (i) creatinine of >114 µmol/L, (ii) a urine output of <1 mL/kg/h, and/or (iii) the requirement for ultrafiltration or hemodialysis; and (6) coagulopathy was defined as (i) requiring chest exploration due to bleeding, (ii) a prothrombin or partial thromboplastin time >3× the normal value, and/or (iii) >30 mL/kg of blood products during the first 24 h postoperative period. We also calculated their Pediatric Logistic Organ Dysfunction-2 (PELOD-2) scores to assess organ dysfunction [57]. Of note, the CPB circuit was routinely primed with one unit of packed red blood cells (pRBCs) and one unit of fresh frozen plasma (FFP), maintaining a target hematocrit level greater than 30%.

Blood samples were collected from the patients through existing central venous catheters. Once the blood samples were received, they were subjected to centrifugation at 1000× *g* for 10 min to obtain plasma. The plasma was immediately stored at −80 °C.

### 4.2. Plasma Collection and EV Isolation

The blood was obtained at the following four time points; (1) immediately after the induction of anesthesia as a baseline sample (T0); (2) in the rewarming phase during CPB (T1); (3) upon admission to the ICU (T2); and (4) on postoperative day 1 (T3). The T0, T2, and T3 plasma was collected from whole blood, and then EVs in the plasma were isolated using the exoEasy Midi kit (Qiagen, Hilden, Germany) per the company’s protocol. EV purification was verified by probing the expression of EV markers (Golgi matrix protein 130 (GM130), CD63, CD81, epithelial cellular adhesion molecule (EpCAM), annexin A5 (ANXA5), tumor susceptibility gene 101 (TSG101), flotillin 1 (FLOT1), intercellular adhesion molecule 1 (ICAM1), and ALG-2-interacting protein X (ALIX)) using the Exo-Check Exosome Antibody Array Kit, Human (System Biosciences, Palo Alto, CA, USA). In addition, the particle size was determined by performing a nanoparticle tracking analysis (NTA) using the ZetaView equipment (Particle Metrix, Ammersee, Germany). The manufacturer’s default software settings for measuring EVs, liposomes, or nanospheres were selected, as applied by us previously [58]. The captured videos were analyzed using the in-built ZetaView Software v.8.05.05 SP2.

### 4.3. EV RNA Extraction, RNA Library Preparation, and Small RNA-Sequencing

The miRNeasy kit (Qiagen, Hilden, Germany) was used to extract the total RNA, including small RNAs. The RNA yield was measured using the Qubit™ microRNA Assay Kit (Thermo Fisher Scientific, Waltham, MA, USA). The RNA’s size distribution and quality were assessed using the Bioanalyzer Small RNA Analysis kit (Agilent Technologies, Santa Clara, CA, USA). Small RNA libraries were constructed using the NEBNext Small RNA Library Prep Set for Illumina (New England Biolabs, Ipswich, MA, USA), as previously used by us [58]. The generated libraries were cleaned up using AMPure XP Beads (Beckman Coulter, Brea, CA, USA) and quantified using the Qubit™ dsDNA HS Assay (Thermo Fisher Scientific) and the Bioanalyzer High Sensitivity DNA Analysis kit (Agilent Technologies). Sequencing was performed on the HiSeq4000 platform (Illumina, San Diego, CA, USA) using the High Output Kit v2.5 and 50-base single reads.

### 4.4. Bioinformatic Analysis

The Galaxy platform was utilized to map the sequencing reads using MiRDeep2 Mapper (version 2.0.0.8.1) to a human reference genome (Homo sapiens: hg38), followed by quantification of the miRNA count with MiRDeep2 Quantifier (version 2.0.0) using miRNA hairpins and mature miRNA sequences retrieved from the miRbase database [59]. DESeq2 (version 2.11.40.8) was used to perform the differential expression analysis. TAM 2 was used to predict the miRNA’s functions, associated diseases, tissue specificity, and families using a cut-off for the false discovery rate (FDR) < 0.05. TAM 2.0 [60], miRTargetLink v.2.0 [61], and miRwalk [62] were used to predict the miRNA gene targets. The BioPlanet and KEGG databases were utilized to curate the pathways of the target genes. The MiEAA v.2.0 platform [63] was used for the miRNA set enrichment analysis. Feature selection implemented using a cross-validation method was performed using R packages (caret v6.0.94 and mblench v2.1.3). Due to technical issues, one of the T0 samples was removed.

### 4.5. Proteomic Sample Preparation

Proteomic samples were prepared as previously reported by us [29]. In brief, plasma samples diluted with urea lysis buffer (9 M urea, 50 mM ammonium bicarbonate with Complete™ Protease Inhibitor Cocktail (Roche, Penzberg, Germany)) were centrifuged at 21,000× *g* for 10 min. Then, the proteins in the supernatant were reduced and alkylated using 10 mM Tris(2-carboxyethyl) phosphine hydrochloride (TCEP-HCl) (Pierce, Appleton, WI, USA) and 10 mM iodoacetamide (VWR, Radnor, PA, USA), followed by quenching with 20 mM cysteine for 30 min. Each sample was diluted to 1.5 M urea with water and digested using trypsin at an enzyme-to-substrate ratio of 1:60 (*w*/*w*) (Promega, Madison, WI, USA).

### 4.6. Nano-HPLC-MS/MS Analysis

The digested peptides were desalted using C18 stage tips, in a similar way to that previously described [29,64], and analyzed at the Center for Metabolomics and Proteomics (CMSP) at the University of Minnesota. Briefly, the desalted tryptic peptides were reconstituted in HPLC sample buffer (97.99:2:0.01, water: acetonitrile (ACN):formic acid (FA)) and loaded onto a self-packed reversed-phase capillary HPLC column (ReproSil-Pur Basic C18aq, with a 40 cm length × a 100 µm internal diameter, a 1.9 µm particle size, and a 120 Å pore size). The peptides were analyzed on a nano-HPLC-MS system incorporating the UltiMate™ 3000 RSLCnano System and an Orbitrap Fusion mass spectrometer (Thermo Fisher). The flowrate profile was 400 nL/min from 0 to 2 min, 315 nL/min from 2.5 to 90 min, and 400 nL/min from 90 to 92 min. The Orbitrap Fusion mass spectrometer (Thermo Fisher) was operated using a full scan range of 380–1580 *m*/*z* at 120,000 resolutions (at 200 *m*/*z*) and the MS/MS analysis acquiring the top 12 precursor ions in data-dependent acquisitions (charge states from 2 to 6) with an isolation window of 1.6 *m*/*z* and the HCD fragmentation energy at 35% in the linear ion trap.

### 4.7. Proteomic Data Analysis

A quantitative analysis of the mass spectrometry data was performed using MaxQuant software (version 1.5.3.12) [65]. The data were searched against a human proteome database (UP000005640.fasta) concatenated with a common contaminant database from MaxQuant and filtered at a 1% FDR for both peptide and protein identification through the reverse target–decoy strategy. A maximum of 1 missing cleavage was allowed for trypsin as the proteolytic enzyme for the database search. The maximum number of modifications per peptide was set to 3. Carbamidomethylation on cysteine was set as a fixed modification, and protein *N*-terminal acetylation and methionine oxidation were set as variable modifications. A label-free quantification analysis (LFQ) was applied for the relative quantification of protein abundance [66]. A pathway analysis was performed as described in the miRNA analysis section.

### 4.8. The Double-Stranded DNA (dsDNA) Assay

Serum dsDNA levels were quantified using a fluorescent nucleic acid stain, Quant-iT PicoGreen (Invitrogen), according to the manufacturer’s protocol. Blood samples were prepared based on the previous study [67] to detect an estimated plasma DNA range from 50 to 200 ng/mL. Fluorescence was excited at 480 nm, and the intensity of the emission was detected at 520 nm. Standard curves were generated for the calculation of the dsDNA concentrations.

### 4.9. Complement Activity Assays

The residual complement activity of classic, alternative, and lectin pathways in the blood was tested using the Wielab complement screen kit (IBL; Hamburg, Germany) per the company’s recommendations.

### 4.10. Statistical Analysis

The data are shown as means ± SDs. A one-way ANOVA with Bonferroni’s post hoc analysis and Student’s unpaired *t*-test were used to assess statistical significance with *p* values * < 0.05, ** < 0.005, and *** < 0.001.

## Figures and Tables

**Table 1:** Patient demographic data in the two study groups.

	Organ Dysfunction (OD)	Non-Organ Dysfunction (NOD)	*p* Value
Age (mo)	5.0 ± 5.1	5.9 ± 4.7	0.70
Male (%)	36% (4/11)	44% (4/9)	0.73
Weight (kg)	5.4 ± 3.1	6.3 ± 2.8	0.52
Salvaged RBCs (mL/kg)	32.7 ± 15.2	16.3 ± 11.1	0.015 *
Platelets (mL/kg)	26.7 ± 15.7	10.9 ± 9.4	0.017 *
Cryoprecipitate (mL/kg)	7.7 ± 9.1	4.1 ± 6.4	0.32
OP time (min)	470.2. ± 89.9	329.9 ± 134.0	0.012 *
CPB time (min)	258.0 ± 64.7	164.9 ± 78.6	0.009 *
X-clamp time (min)	138.3 ± 55.5	87.3± 56.9	0.06
Circulatory arrest (%)	36% (4/11)	33% (3/9)	0.89
Regional perfusion (%)	36% (4/11)	33% (3/9)	0.89
MV duration (h)	300.9 ± 296.2	50.5 ± 29.3	0.02 *
ICU stay (h)	369.0 ± 299.4	73.8 ± 58.1	0.001 *
LOS (d)	36.2 ± 22.6	10.0 ± 6.1	0.004 *
PELOD-2 POD#0	9.3 ± 2.4	6.2 ± 1.7	0.005 *
PELOD-2 POD#1	5.0 ± 1.7	3.6 ± 2.4	0.13
PELOD-2 POD#2	5.9 ± 1.6	3.1 ± 2.4	0.007 *

Data are expressed as means  ±  standard deviations. Significant differences between study groups are indicated by * (*p*  ≤  0.05). Abbreviations: OP, operation; X-clamp, aortic cross clamp; CPB, cardiopulmonary bypass: MV, mechanical ventilation: LOS, length of hospital stay: PELOD, Pediatric Logistic Organ Dysfunction (severity score): POD, postoperative day.

## Data Availability

The raw sequencing data can be made available on reasonable request to the corresponding author.

## Supplementary Materials

The following supporting information can be downloaded at: https://www.mdpi.com/article/10.3390/ijms26083837/s1.

## Abbreviations

CHD, congenital heart disease; KID, the Kids’ Inpatient Database; STS, the Society of Thoracic Surgeons; CPB, cardiopulmonary bypass; EV, extracellular vesicle; GM130, Golgi matrix protein 130; EpCAM, epithelial cellular adhesion molecule; ANXA5, annexin A5; TSG101, tumor susceptibility gene 101; FLOT1, flotillin 1; ICAM-1, intercellular adhesion molecule 1; ALIX, ALG-2-interacting protein X; NTA, nanoparticle tracking analysis; FDR, false discovery rate; ACN, acetonitrile; FA, formic acid; LFQ, label-free quantification analysis; dsDNA, double-stranded DNA; OD, organ dysfunction; NOD, non-organ dysfunction; RBC, red blood cell; pRBC, packed red blood cell; FFP, fresh frozen plasma; MV, mechanical ventilation; PELOD, Pediatric Logistic Organ Dysfunction; POD, postoperative day; UMAP, Uniform Manifold Approximation and Projection; PCA, principal component analysis; DE, differentially expressed; DAMP, damage-associated molecular pattern; NET, neutrophil extracellular trap; MBL, mannose-binding lectin; MASP1, mannose-binding lectin-associated serine protease 1; FCN3, ficolin 3; TNC, tenascin C; HSPG2, heparan sulfate proteoglycan 2; VWF, von Willebrand factor; SELENOP, selenoprotein P; APOA1, apolipoprotein A1; CRP, C-reactive protein; UTR, untranslated region; AMI, acute myocardial infarction; ASC, acute coronary syndrome; TNFSF8, TNF superfamily member 8; SIRS, systemic inflammatory response syndrome; MODS, multi-organ dysfunction.
