# Supplementary material for: The Role of Extracellular-Vesicle-Derived miRNAs in Postoperative Organ Dysfunction in Neonates and Infants Undergoing Congenital Cardiac Surgery: An Exploratory Study
Source: Int J Mol Sci. 2025 Apr 18;26(8):3837. doi: 10.3390/ijms26083837 (PMC12028143; doi:10.3390/ijms26083837)
Supplement: Supplementary file 1 [file ijms-26-03837-s001.zip › ijms-3486889-supplementary.pdf]

**Supplementary Data**

Title: The role of extracellular vesicle miRNAs in postoperative organ dysfunction in neonates and infants undergoing pediatric congenital cardiac surgery

Fahd Alhamdan, Wiriya Maisat, LeeAnn Higgs, Yue Chen, Juan Ibla, Koichi Yuki

**Supplementary Figures**

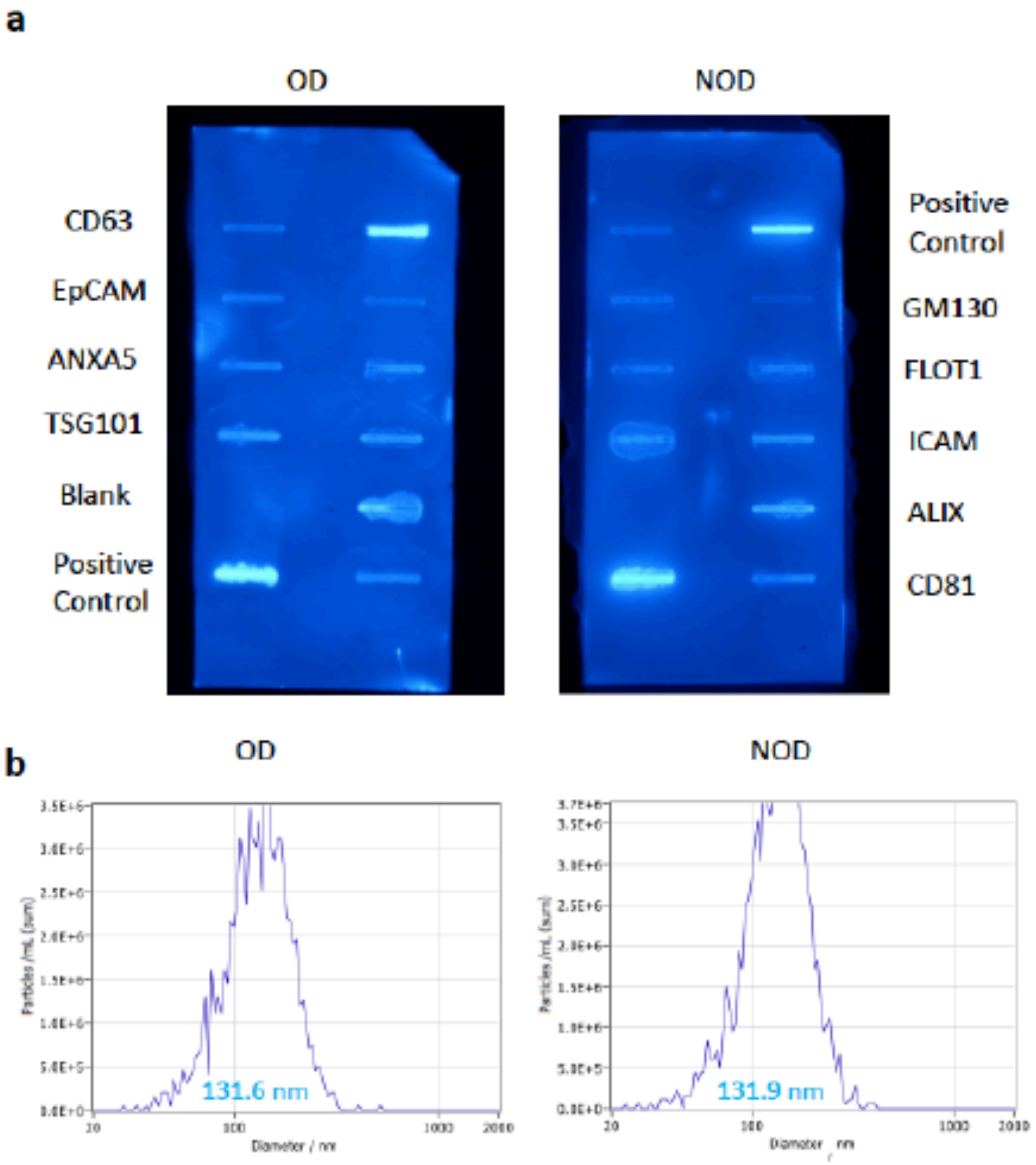


**Supplementary Figure S 1: Characterization of EVs size and protein markers. a**, Raw membrane visualization of the EV protein markers of both OD and NOD groups. **b**, Nanoparticle Tracking Analysis (NTA) measure the particle concentration and EV size of both OD and NOD groups.

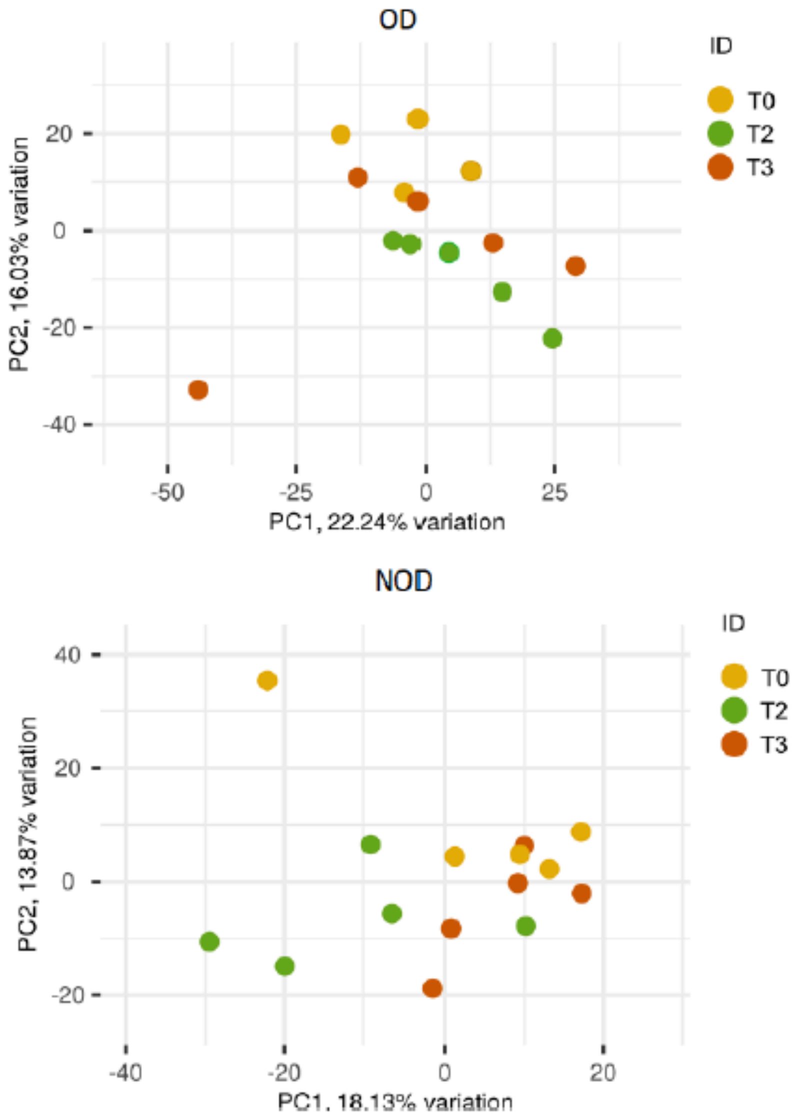


**Supplementary Figure S2: Distribution of EV miRNAs at the three time points.** Principal Component Analysis (PCA) of the expression profiles of plasma EV miRNAs in **a**, OD and **b**, NOD groups at the three time points.

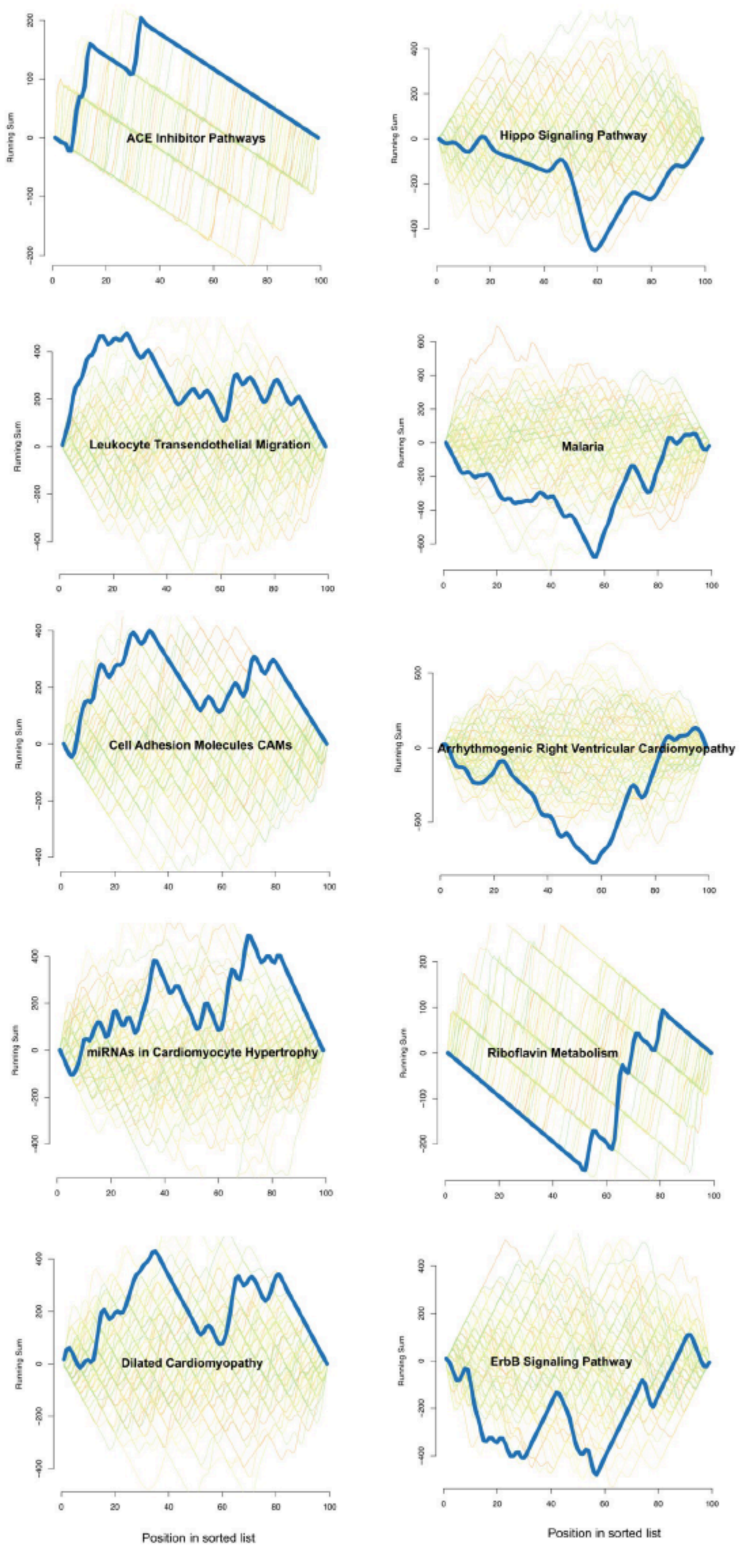


**Supplementary Figure S3: Enriched biological pathways for unique T0 EV miRNAs.** Gene set enrichment analysis (GSEA) plots of upregulated or downregulated biological pathways associated with unique T0 EV miRNAs. Simulated background distributions (green to orange lines) and actual enrichment of the respective pathways (blue line).

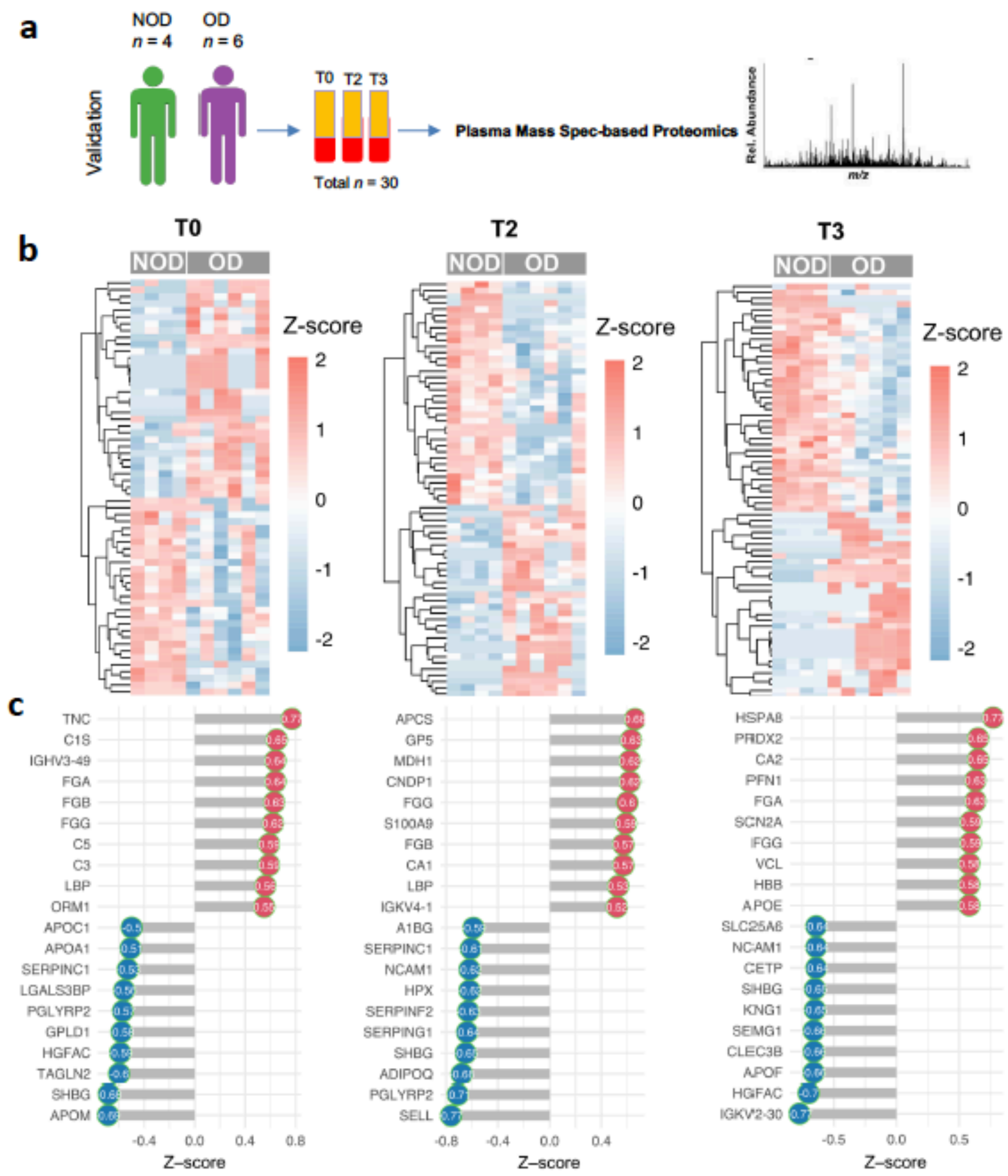


**Supplementary Figure S4: Expression profiles of plasma proteins during pediatric congenital cardiac surgery from a second cohort**. **a**, Schematic representation of the study design. Organ dysfunction: OD (*n* = 6) and non-organ dysfunction: NOD (*n* = 4). T0: after anesthesia induction (baseline), T2: upon admission to the ICU, and T3: on postoperative day 1. Heatmaps depicting the expression levels of significant plasma proteins of OD versus NOD pairwise comparisons at the three time points. **b**, Dot plots of the top 10 upregulated and downregulated proteins according to their $\log_2$(FC) scores for each time point.

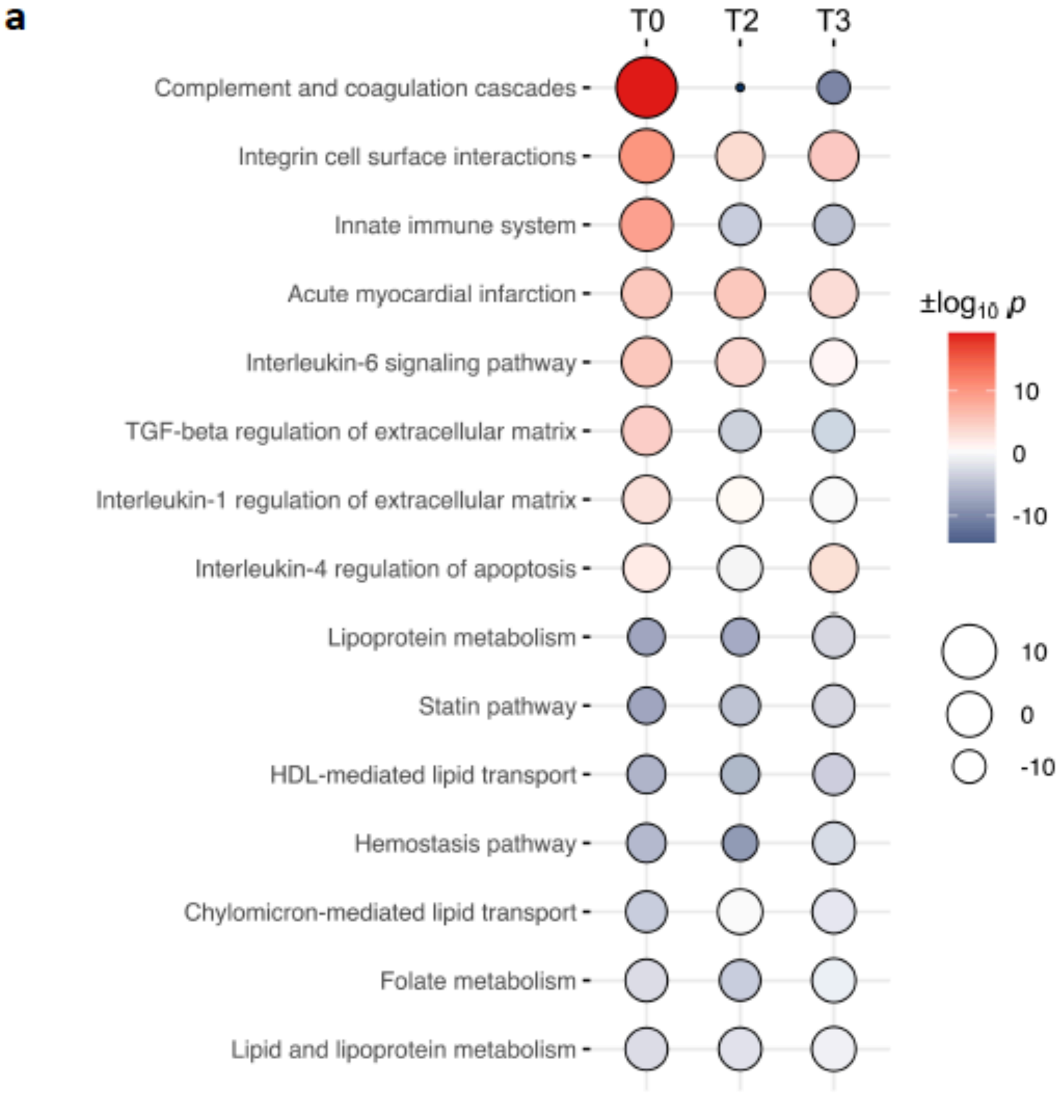


**Supplementary Figure S5: Top 15 unique and shared biological pathways for the plasma proteins in the three time points during pediatric congenital cardiac surgery**. Dot plot exhibiting the top 15 significant upregulated (red) or downregulated (blue) biological pathways ($p < 0.05$) for the plasma proteins in OD versus NOD for the three time points.

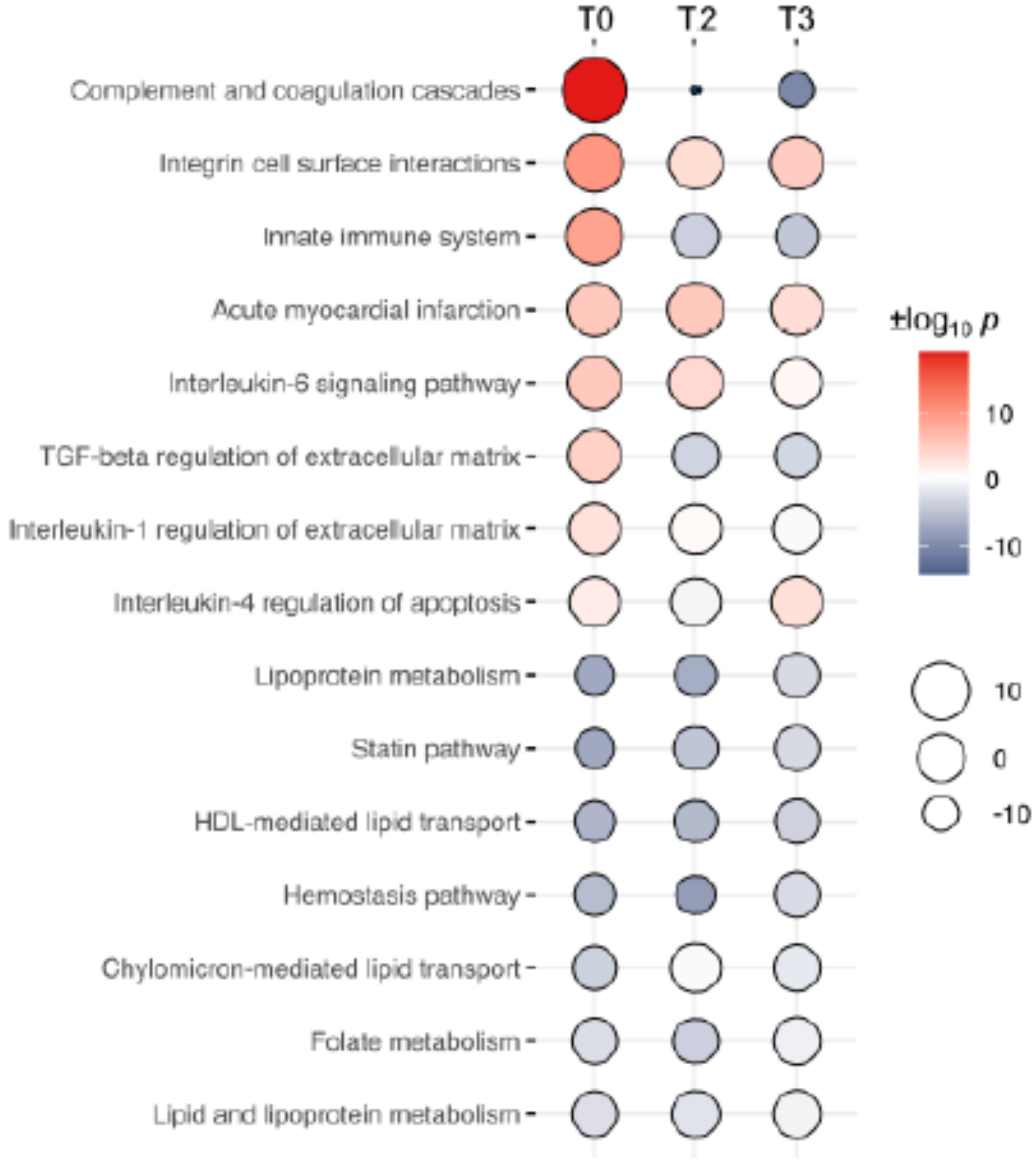


**Supplementary Figure S6: Enriched biological pathways for plasma proteins at T0.** Bar plot of the top 5 significant upregulated or downregulated biological pathways ($p < 0.05$) for the plasma proteins in OD versus NOD at T1.

## Supplementary Tables

**Supplementary Table S1. Age, gender, surgery type, and type of organ dysfunction in the miRNomics clinical cohort**

| Patient ID | Age (m) | Gender | Surgery type | OD type |
|---|---|---|---|---|
| **OD1** | 12 | F | PVS repair | Low cardiac output |
| **OD2** | 12 | F | Double switch | Respiratory failure, low cardiac output |
| **OD3** | 4 | F | PVS repair | Respiratory failure |
| **OD4** | 0.1 | F | Norwood procedure | Respiratory failure, low cardiac output |
| **OD5** | 10 | M | DORV repair | Respiratory failure, low cardiac output |
| | | | | |
| **NOD1** | 0.1 | F | Aortic arch & VSD repair | None |
| **NOD2** | 12 | F | VSD repair | None |
| **NOD3** | 12 | F | VSD repair | None |
| **NOD4** | 8 | M | Norwood procedure | None |
| **NOD5** | 1 | M | Aortic arch and VSD repair | None |

Abbreviations: OD: Organ dysfunction; NOD: Non-Organ dysfunction; M: Male; F: Female m: month; PVS: Pulmonary Vein Stenosis; DORV: Double Outlet Right Ventricle; VSD: Ventricular septal defect.

## Supplementary Table S2. Differentially expressed miRNAs in OD versus NOD at T0, T2, or T3

| T0 | LFC | P-adj | T2 | LFC | P-adj | T3 | LFC | P-adj |
|---|---|---|---|---|---|---|---|---|
| miR-151a-3p | 2.55 | 2.19E-07 | miR-4787-5p | -2.52 | 0.00031159 | miR-199a-5p | 2.14 | 0.00014558 |
| miR-326 | 2.69 | 2.19E-07 | miR-6789-5p | -2.46 | 0.00123416 | miR-146a-5p | 2.24 | 0.00102482 |
| miR-335-5p | 2.63 | 2.19E-07 | miR-3665 | -2.22 | 0.00234442 | miR-224-5p | 3.25 | 0.00102482 |
| miR-758-3p | 2.8 | 6.24E-07 | miR-6768-5p | -1.37 | 0.00234442 | miR-543 | 2.55 | 0.00102482 |
| miR-199a-5p | 3 | 7.92E-07 | miR-2861 | -2.77 | 0.00429081 | miR-3163 | -3.65 | 0.0016817 |
| miR-330-3p | 2.77 | 8.58E-07 | miR-4734 | -1.19 | 0.00429081 | miR-4482-5p | -4.08 | 0.00174154 |
| miR-146a-5p | 2.43 | 9.55E-07 | miR-8053 | -2.27 | 0.00429081 | miR-432-5p | 2.97 | 0.00208305 |
| miR-221-3p | 2.46 | 3.20E-06 | miR-1282 | -2.12 | 0.00476615 | miR-151a-3p | 1.91 | 0.0022558 |
| miR-28-5p | 2.3 | 6.48E-06 | miR-3663-3p | -2.26 | 0.00476615 | miR-379-3p | 3 | 0.0022558 |
| miR-199a-3p | 2.62 | 6.93E-06 | miR-1469 | -1.82 | 0.00575984 | miR-379-5p | 3.21 | 0.00247631 |
| miR-199b-3p | 2.61 | 6.93E-06 | miR-12121 | -2.44 | 0.00580037 | miR-28-5p | 1.89 | 0.00262835 |
| miR-584-5p | 2.2 | 7.86E-06 | miR-4785 | -2.05 | 0.00580037 | miR-493-5p | 3 | 0.00319526 |
| miR-223-5p | 2.02 | 1.34E-05 | miR-4466 | -1.85 | 0.00601226 | miR-126-5p | 1.69 | 0.00359869 |
| miR-21-5p | 2.25 | 1.51E-05 | miR-7704 | -2.62 | 0.00615014 | miR-221-3p | 1.65 | 0.00431572 |
| miR-339-5p | 2.26 | 1.51E-05 | miR-548d-5p | -1.35 | 0.00682953 | miR-433-3p | 2.2 | 0.00431572 |
| let-7i-5p | 1.93 | 1.74E-05 | miR-122-5p | 2.89 | 0.00690792 | miR-483-5p | 2.48 | 0.00431572 |
| miR-191-5p | 2.52 | 1.74E-05 | miR-1298-3p | -1.83 | 0.00975487 | miR-21-5p | 1.94 | 0.0052737 |
| miR-221-5p | 2.12 | 1.74E-05 | miR-2277-3p | -1.8 | 0.00975487 | miR-548n | -2.98 | 0.00530979 |
| miR-374b-5p | 2.48 | 1.92E-05 | miR-3178 | -2.36 | 0.00975487 | miR-148b-3p | 1.81 | 0.00546099 |
| miR-148b-3p | 2.26 | 3.01E-05 | miR-3195 | -2.57 | 0.00975487 | miR-369-5p | 2.88 | 0.00546099 |
| miR-152-3p | 2.26 | 3.01E-05 | miR-34a-5p | -2.07 | 0.00975487 | miR-7849-3p | -2.65 | 0.00546099 |
| miR-26a-5p | 2.18 | 3.01E-05 | miR-10394-3p | -1.51 | 0.01047996 | miR-548ab | -2.91 | 0.00784809 |
| miR-1185-1-3p | 2.12 | 3.15E-05 | miR-1237-5p | -1.68 | 0.01383994 | miR-200b-5p | -1.97 | 0.01042573 |
| miR-98-5p | 2.08 | 4.08E-05 | miR-3935 | -2.87 | 0.01402486 | miR-584-5p | 1.62 | 0.01445857 |
| miR-654-3p | 2.36 | 4.18E-05 | miR-548at-5p | -2.32 | 0.01402486 | miR-136-3p | 2.61 | 0.01463955 |
| miR-543 | 3.43 | 6.34E-05 | miR-6084 | -1.87 | 0.01507749 | miR-221-5p | 1.93 | 0.01463955 |
| miR-148a-3p | 1.74 | 6.82E-05 | miR-6505-5p | -2.55 | 0.0151757 | miR-26a-5p | 1.64 | 0.01463955 |
| miR-410-3p | 2.47 | 8.56E-05 | miR-7108-5p | -1.45 | 0.01915048 | miR-361-3p | 1.46 | 0.01463955 |
| miR-151b | 2.22 | 9.47E-05 | miR-5787 | -1.62 | 0.02827853 | miR-363-3p | 1.46 | 0.01463955 |
| miR-134-5p | 2.53 | 0.00013484 | miR-4707-5p | -1.07 | 0.03252566 | miR-381-3p | 2.74 | 0.01463955 |
| miR-744-5p | 2.27 | 0.00013484 | miR-6724-5p | -1.91 | 0.03465005 | miR-382-3p | 2.5 | 0.01463955 |
| miR-103a-3p | 2.19 | 0.00013994 | miR-203a-3p | -1.29 | 0.03648574 | miR-411-3p | 2.74 | 0.01463955 |
| miR-337-3p | 3.02 | 0.00013994 | miR-181b-3p | -1.21 | 0.03935346 | miR-889-3p | 2.58 | 0.01463955 |
| miR-628-5p | 2.3 | 0.00015243 | miR-3181 | -1.3 | 0.03935346 | miR-30c-5p | 1.5 | 0.0187555 |
| miR-224-5p | 2.4 | 0.00016657 | miR-6088 | -1.63 | 0.03935346 | miR-4800-5p | 1.41 | 0.0187555 |

| | | | | | | | | |
|---|---|---|---|---|---|---|---|---|
| miR-139-3p | 1.73 | 0.00020817 | miR-638 | -1.81 | 0.03935346 | miR-126-3p | 1.53 | 0.01953758 |
| miR-223-3p | 2.26 | 0.00020817 | miR-6727-5p | -1.5 | 0.04097408 | miR-1266-5p | -2.43 | 0.01953758 |
| miR-30d-5p | 1.84 | 0.00020817 | miR-1307-3p | -1.31 | 0.04755704 | miR-146b-5p | 1.7 | 0.01953758 |
| miR-24-3p | 1.91 | 0.00029712 | | | | miR-30d-5p | 1.3 | 0.01953758 |
| miR-1301-3p | 2.27 | 0.00031646 | | | | miR-382-5p | 2.36 | 0.01953758 |
| miR-652-3p | 1.98 | 0.00031646 | | | | miR-495-3p | 2.24 | 0.01953758 |
| miR-942-5p | 1.9 | 0.0003513 | | | | miR-6073 | 2.37 | 0.01953758 |
| miR-30e-3p | 1.59 | 0.00036498 | | | | let-7i-5p | 1.62 | 0.0203562 |
| miR-411-5p | 2.22 | 0.00038543 | | | | miR-654-3p | 2.33 | 0.02133774 |
| miR-12126 | -2.84 | 0.00043838 | | | | miR-548ay-5p | 1.41 | 0.02212513 |
| miR-126-5p | 1.78 | 0.00050709 | | | | miR-223-5p | 1.62 | 0.02290704 |
| miR-99b-5p | 1.72 | 0.00053108 | | | | miR-410-3p | 2.4 | 0.02290704 |
| miR-140-3p | 1.77 | 0.00054034 | | | | miR-340-5p | 1.73 | 0.02645222 |
| miR-493-3p | 2.57 | 0.00054034 | | | | miR-223-3p | 1.61 | 0.02819903 |
| miR-4446-3p | 1.88 | 0.00055994 | | | | miR-148a-3p | 1.63 | 0.02844135 |
| miR-222-3p | 2.09 | 0.00058003 | | | | miR-199a-3p | 1.61 | 0.02844135 |
| miR-126-3p | 1.47 | 0.00059127 | | | | miR-340-3p | 1.82 | 0.02844135 |
| miR-432-5p | 2.43 | 0.00064133 | | | | miR-199b-3p | 1.6 | 0.0289629 |
| miR-127-3p | 2.41 | 0.00065786 | | | | miR-505-3p | -1.35 | 0.02980434 |
| miR-340-3p | 2.2 | 0.00066896 | | | | miR-548ae-5p | 1.37 | 0.02980434 |
| miR-423-3p | 2.14 | 0.00066896 | | | | miR-892b | -2.25 | 0.02980434 |
| miR-889-3p | 2.33 | 0.00073651 | | | | miR-98-5p | 1.47 | 0.02980434 |
| miR-379-5p | 2.44 | 0.00076582 | | | | miR-4726-3p | -1.82 | 0.02987242 |
| miR-409-5p | 1.69 | 0.00077825 | | | | miR-3613-5p | 1.61 | 0.03042193 |
| miR-411-3p | 2.35 | 0.00079975 | | | | miR-4291 | -1.78 | 0.03414901 |
| let-7f-5p | 1.4 | 0.00083353 | | | | miR-4690-3p | -1.85 | 0.03414901 |
| miR-6125 | -3.36 | 0.00083353 | | | | miR-5698 | -2.54 | 0.03414901 |
| miR-654-5p | 1.89 | 0.00083353 | | | | miR-103a-3p | 1.34 | 0.03615646 |
| let-7d-5p | 1.75 | 0.00084649 | | | | miR-134-5p | 2.3 | 0.03615646 |
| miR-128-3p | 1.94 | 0.00092201 | | | | miR-539-5p | 2.2 | 0.03615646 |
| miR-1184 | -2.92 | 0.00097514 | | | | miR-19b-3p | 1.24 | 0.03836508 |
| miR-340-5p | 1.99 | 0.00099196 | | | | miR-548ad-5p | 1.4 | 0.04001727 |
| miR-19a-3p | 1.87 | 0.00124398 | | | | miR-142-3p | 1.66 | 0.04071009 |
| miR-382-5p | 2.42 | 0.00128557 | | | | miR-191-5p | 1.38 | 0.04071009 |
| miR-184 | -2.21 | 0.00131172 | | | | miR-494-3p | 1.67 | 0.04071009 |
| miR-425-5p | 1.88 | 0.00131172 | | | | miR-6846-5p | -1.79 | 0.04071009 |
| miR-381-3p | 2.19 | 0.00132123 | | | | miR-17-5p | 1.49 | 0.04110746 |
| miR-28-3p | 1.52 | 0.00140692 | | | | miR-181a-5p | 0.93 | 0.04193833 |

| | | | | | | | | |
|---|---|---|---|---|---|---|---|---|
| miR-374a-5p | 1.97 | 0.00140692 | | | | miR-1185-1-3p | 1.74 | 0.04284866 |
| miR-335-3p | 2.53 | 0.00145748 | | | | miR-186-5p | 1.24 | 0.04284866 |
| miR-487b-3p | 2.26 | 0.00150789 | | | | miR-625-3p | 2.05 | 0.04284866 |
| miR-1911-3p | -2.33 | 0.0021253 | | | | miR-561-5p | -2.28 | 0.04493465 |
| miR-6852-5p | 1.62 | 0.0021253 | | | | miR-335-5p | 1.82 | 0.04573761 |
| miR-151a-5p | 2.01 | 0.00214602 | | | | miR-34c-5p | 2.27 | 0.04573761 |
| miR-17-5p | 1.92 | 0.00221273 | | | | miR-30e-5p | 1.34 | 0.04688616 |
| miR-136-3p | 2.14 | 0.00242922 | | | | miR-5580-5p | 2.61 | 0.04688616 |
| miR-140-5p | 1.74 | 0.00257479 | | | | miR-26b-5p | 1.36 | 0.04761931 |
| miR-30e-5p | 1.77 | 0.00270303 | | | | miR-7106-3p | -1.99 | 0.04761931 |
| miR-1271-5p | 2.07 | 0.00285282 | | | | miR-7843-3p | -2.16 | 0.04761931 |
| miR-26b-5p | 1.73 | 0.00287028 | | | | miR-1250-5p | -1.46 | 0.04922957 |
| miR-18a-5p | 1.64 | 0.00302908 | | | | miR-409-3p | 1.93 | 0.05041783 |
| miR-409-3p | 2.28 | 0.00302908 | | | | | | |
| miR-671-3p | 2.02 | 0.00333333 | | | | | | |
| miR-625-3p | 1.59 | 0.00334711 | | | | | | |
| miR-495-3p | 2.25 | 0.00336803 | | | | | | |
| miR-328-3p | 2.9 | 0.00338986 | | | | | | |
| miR-645 | -2.3 | 0.00350818 | | | | | | |
| miR-493-5p | 2.46 | 0.00377572 | | | | | | |
| miR-539-3p | 2.13 | 0.00409749 | | | | | | |
| miR-2355-3p | 1.68 | 0.00439056 | | | | | | |
| miR-4433b-5p | 2.66 | 0.00465295 | | | | | | |
| miR-142-3p | 1.84 | 0.0048619 | | | | | | |
| miR-484 | 1.78 | 0.00554121 | | | | | | |
| miR-628-3p | 1.68 | 0.00563321 | | | | | | |
| miR-4485-3p | 2.47 | 0.00703899 | | | | | | |
| miR-30c-5p | 1.51 | 0.00766971 | | | | | | |
| miR-6073 | 2.43 | 0.00767065 | | | | | | |
| miR-7703 | -2.46 | 0.00767065 | | | | | | |
| miR-548ad-5p | -1.92 | 0.0078963 | | | | | | |
| miR-1273h-3p | 2.04 | 0.00798878 | | | | | | |
| miR-146b-5p | 1.27 | 0.00820138 | | | | | | |
| miR-433-3p | 1.67 | 0.00827274 | | | | | | |
| miR-452-5p | 1.4 | 0.00844159 | | | | | | |
| miR-1277-5p | 2.12 | 0.00881146 | | | | | | |
| miR-196b-5p | 1.76 | 0.00939065 | | | | | | |
| miR-139-5p | 1.12 | 0.01012774 | | | | | | |

| | | | | | | | | |
|---|---|---|---|---|---|---|---|---|
| miR-423-5p | 1.38 | 0.01062122 | | | | | | |
| miR-320a-3p | 1.18 | 0.01226067 | | | | | | |
| miR-877-5p | 1.55 | 0.01297922 | | | | | | |
| miR-329-3p | 1.98 | 0.01426089 | | | | | | |
| let-7g-5p | 1.26 | 0.01622464 | | | | | | |
| miR-185-3p | 1.65 | 0.01622464 | | | | | | |
| miR-20a-5p | 1.71 | 0.0171597 | | | | | | |
| let-7a-5p | 0.9 | 0.01802845 | | | | | | |
| miR-421 | 1.61 | 0.01854264 | | | | | | |
| miR-548j-5p | 1.9 | 0.01926416 | | | | | | |
| miR-323a-3p | 1.91 | 0.02007436 | | | | | | |
| miR-323b-3p | 1.74 | 0.02011444 | | | | | | |
| miR-4682 | -2.12 | 0.020426 | | | | | | |
| miR-485-3p | -1.31 | 0.02042927 | | | | | | |
| miR-5004-3p | -1.64 | 0.02052708 | | | | | | |
| miR-93-5p | 1.32 | 0.02198452 | | | | | | |
| miR-548ay-5p | -1.71 | 0.02262708 | | | | | | |
| miR-136-5p | 1.89 | 0.02357169 | | | | | | |
| miR-197-3p | 1.81 | 0.02357169 | | | | | | |
| miR-382-3p | 1.75 | 0.02426019 | | | | | | |
| miR-8053 | -2.57 | 0.02453007 | | | | | | |
| miR-30b-5p | 1.43 | 0.02459204 | | | | | | |
| miR-143-3p | 1.17 | 0.02779495 | | | | | | |
| miR-378e | -1.12 | 0.0298432 | | | | | | |
| miR-378d | -1.07 | 0.03284043 | | | | | | |
| miR-656-3p | 1.93 | 0.03284043 | | | | | | |
| miR-4433b-3p | 1.42 | 0.03334981 | | | | | | |
| miR-4703-3p | -1.95 | 0.03430038 | | | | | | |
| miR-203a-3p | -1.24 | 0.03448498 | | | | | | |
| miR-548ae-5p | -1.63 | 0.03580586 | | | | | | |
| miR-23a-3p | 1.36 | 0.03624035 | | | | | | |
| miR-548b-5p | -1.47 | 0.03739397 | | | | | | |
| miR-181d-5p | 1.06 | 0.03780413 | | | | | | |
| miR-199b-5p | 1.19 | 0.03780413 | | | | | | |
| miR-4676-3p | -2.15 | 0.03780413 | | | | | | |
| miR-631 | -1.23 | 0.03834696 | | | | | | |
| miR-98-3p | 2.41 | 0.04032236 | | | | | | |
| miR-6800-3p | -2.07 | 0.04142732 | | | | | | |
| miR-5001-5p | -0.95 | 0.04233475 | | | | | | |

| | | | | | | | | | |
|---|---|---|---|---|---|---|---|---|---|
| miR-424-3p | 1.24 | 0.04253383 | | | | | | | |
| miR-374a-3p | 1.13 | 0.04313409 | | | | | | | |
| miR-379-3p | 1.73 | 0.04460512 | | | | | | | |
| miR-361-3p | 1.08 | 0.04922388 | | | | | | | |
| miR-1257 | -1.81 | 0.04984014 | | | | | | | |

**Supplementary Table S3. Age, gender, surgery type, and type of organ dysfunction in the Proteomics clinical cohort**

| Patient ID | Age (m) | Gender | Surgery type | OD type |
|---|---|---|---|---|
| **OD1** | 0.4 | M | TAPVR repair | Multi organ failure/death |
| **OD2** | 0.3 | M | TOF repair | Respiratory failure, low cardiac output |
| **OD3** | 0.1 | F | Interrupted aortic arch repair | Respiratory failure, low cardiac output |
| **OD4** | 10 | M | DORV repair | Respiratory failure, low cardiac output |
| **OD5** | 6 | F | TOF/CAVC repair | Stroke, respiratory failure |
| **OD6** | 0.2 | F | Interrupted aortic arch repair | Thrombosis |
| **NOD1** | 5 | F | VSD repair | None |
| **NOD2** | 10 | F | Multiple VSD closure | None |
| **NOD3** | 1 | M | VSD repair | None |
| **NOD4** | 4 | M | TOF repair | None |

Abbreviations: OD: Organ dysfunction; NOD: Non-Organ dysfunction; M: Male; F: Female; m: month; TAPVR: Total Anomalous Pulmonary Venous Return; TOF: Tetralogy of Fallot; DORV: Double Outlet Right Ventricle; CAVC: Complete Atrioventricular Canal defect; VSD: Ventricular septal defect.

## Supplementary Table S4. Detected proteins of complement system and its three pathways

| Complement components | Classical pathway | Lectin pathway | Alternative pathway |
|---|---|---|---|
| **MASP2** | C1QA | MASP2 | C8A |
| **C1R** | C1QB | C2 | C8G |
| **C3** | C1QC | C8A | C6 |
| **C5** | C2 | C8G | |
| **C1QA** | C8A | C4A | |
| **C1QB** | C1S | C4B | |
| **C1QC** | C4A | MASP1 | |
| **C9** | C4B | | |
| **C4BPA** | | | |
| **C2** | | | |
| **C8A** | | | |
| **C8G** | | | |
| **C1S** | | | |
| **C4A** | | | |
| **C4B** | | | |
| **C7** | | | |
| **C6** | | | |
| **MASP1** | | | |
| **C1RL** | | | |
